# Supplementary material for: TastepepAI: An artificial intelligence platform for taste peptide de novo design
Source: PLoS Comput Biol. 2025 Oct 16;21(10):e1013602. doi: 10.1371/journal.pcbi.1013602 (PMC12543283; doi:10.1371/journal.pcbi.1013602)
Supplement: S1 File — (ZIP) [file pcbi.1013602.s006.zip › Supplementary Data 2/TaPep41 HPLC.doc]

**HPLC REPORT**

| Sample: | FCl-NJP93344 TaPep41 PD-7 | | | Analyzed date: | 2024-9-21 |
| --- | --- | --- | --- | --- | --- |
| Analyst: | WGJ | | | Reconstitution: | H_2_O:ACN=5:1 |
| Lot. No.: | P240913-MX1172259 | | | | |
| Column: | COSMOSIL Packed Column, 4.6*250mm, 5μm | | | | |
| Solvent A | A: 0.1% Trifluoroacetic Acid in 100% Acetonitrile | | | | |
| Solvent B | B: 0.1% Trifluoroacetic Acid in 100% Water | | | | |
| Gradient: |  | A | B |  | |
|  | 0.0min | 7% | 93% |  | |
|  | 25.0min | 34% | 66% |  | |
|  | 25.1min | 100% | 0% |  | |
|  | 30.0min | Stop | |  | |
| Volume: | 5μl | | |  | |
| Wavelength: | 220nm | | |  | |
| Flow rate: | 1.0ml/min | | |  | |
|  | | | | | |
| ────────────────────────────────────────────────────  Peak Time Conc. Area Height  ────────────────────────────────────────────────────.  1 11.629 0.4814 45374 38632  2 11.717 97.34 9174318 760958  3 12.025 1.436 135390 22309  4 12.408 0.2084 19642 4519  5 12.591 0.5366 50581 8644  Total 100 9425305 835062 | | | | | |
